# The Diagnostic Value of Synovial Fluid Lymphocytes in Gout Patients

**DOI:** 10.1155/2021/4385611

**Published:** 2021-09-15

**Authors:** Qidang Huang, Yukai Huang, Xin Guo, Junming Chen, Zheng Zhong, Yuqi Liu, Weiming Deng, Tianwang Li

**Affiliations:** ^1^Department of Rheumatology and Immunology, Guangdong Second Provincial General Hospital, Guangzhou 510317, China; ^2^The Second School of Clinical Medicine, Southern Medical University, Guangzhou 510515, China

## Abstract

**Objective:**

This study is aimed at investigating the diagnostic value of synovial fluid cell counts in gout patients.

**Methods:**

A total of 185 gout, 64 rheumatoid arthritis (RA), 26 axial spondyloarthritis (axSpA), and 24 osteoarthritis (OA) patients were included in the study. According to serum uric acid (sUA) levels on attack, gout patients were divided into normal sUA gout patients and high sUA gout patients. The laboratory data were recorded. ROC curves were generated to evaluate the diagnostic value of the variables for gout patients and normal sUA gout patients compared with RA, axSpA, and OA patients.

**Results:**

The synovial fluid white blood cell (WBC), peripheral blood mononuclear cell (PBMC), monocyte, polymorphonuclear (PMN), and neutrophil counts in gout patients were higher than those in OA patients (*P* < 0.05). The synovial fluid PBMC and lymphocyte counts in gout patients were lower than those in RA and axSpA patients (*P* < 0.05). ROC curve results showed that the AUC values of lymphocytes and sUA for gout patients were 0.728 and 0.881, respectively, which were higher than those of other variables. The optimal cutoff value of lymphocytes for gout was 1.362, with a Youden index of 0.439, a sensitivity of 83.3%, and a specificity of 60.6%. The AUC values of lymphocytes, sUA, and CRP for normal sUA gout patients were 0.694, 0.643, and 0.700, respectively, which were higher than those of other variables. The optimal cutoff value of lymphocytes for normal sUA gout patients was 1.362, with a Youden index of 0.422, a sensitivity of 81.6%, and a specificity of 60.6%.

**Conclusions:**

The synovial fluid cell counts of gout patients were different from those of RA, axSpA, and OA patients. Synovial fluid lymphocytes had a higher diagnostic value for gout.

## 1. Introduction

Gout is one of the most common types of inflammatory arthritis caused by deposition of monosodium urate (MSU) crystals, with increases in incidence and prevalence in many countries [1, 2]. It has been proposed that resident phagocytic cells can be activated by crystal deposition in clear crystals, which results in a massive influx of leukocytes into the joint [3, 4]. The components of synovial fluid are closely related to the pathogenesis of gout. High levels of cytokines such as interleukin 1 (IL-1*β*), IL-63, IL-85, and tumor necrosis factor *α* (TNF*α*) have been detected in the synovial fluid of gout patients [5–7].

Synovial fluid cell count has long been recognized to have utility in the diagnosis and management of arthritis [8]. The determination of leukocytes in synovial fluid is the most important tool to discriminate inflammatory arthritis from noninflammatory arthritis [9]. A total leukocyte count >2∗10^9^/L is indicative of inflammatory joint diseases, such as gout, rheumatoid arthritis (RA), and axial spondyloarthritis (axSpA). Conversely, a leukocyte count < 2∗10^9^/L points to a noninflammatory origin, such as osteoarthritis (OA) [10, 11]. It has been reported that the number of leukocytes and the percentage of polymorphonuclear (PMN) cells in synovial fluid in the acute phase of gout are higher than those in the remission phase [5]. McCabe et al. found that total synovial fluid leukocytes appeared to be a biomarker for MRI synovitis and may also predict response to treatment in OA patients [12]. Lymphocyte or lymphocyte-derived indices are somehow involved in the processes of many inflammatory conditions, including COVID-19, irritable bowel syndrome, thyroiditis, and frailty [13–16]. The diagnostic value of synovial fluid peripheral blood mononuclear cells (PBMCs), eosinophils, neutrophils, lymphocytes, and monocytes in gout patients compared with RA, axSpA, and OA patients has not yet been described.

Accordingly, we retrospectively collected the synovial fluid cell counts of gout, RA, axSpA, and OA patients and investigated the diagnostic value of synovial fluid cell counts for gout.

## 2. Materials and Methods

### 2.1. Participants' Characteristics

A total of 185 gout patients, 64 RA patients, 26 axSpA patients, and 24 OA patients seen between February 2013 and August 2020 were enrolled in the study. The inclusion criteria were as follows: patients with pain in the knee joints, and synovial fluid cell counts were evaluated by a Sysmex XP-300 (Sysmex Corporation, Kobe, Japan). Gout was diagnosed on the basis of the American College of Rheumatology/European League Against Rheumatism (ACR/EULAR) 2015 criteria. RA was diagnosed on the basis of the ACR/EULAR 2010 criteria. AxSpA was diagnosed using the 2009 ASAS classification criteria for the diagnosis of axSpA. OA was diagnosed on the basis of X-ray findings of reduced medial joint space. According to the serum uric acid (sUA) level during attack, gout patients were divided into a normal sUA gout group (sUA ≤ 420 *μ*M, *n* = 52) and a high sUA gout group (sUA > 420 *μ*M, *n* = 133). The study was approved by the EC office of the Guangdong Second Provincial General Hospital (2021-KZ-131-01). The patient's permission was obtained.

### 2.2. Collection of Laboratory Data

Age, sex, synovial fluid cell counts (WBCs, PBMCs, monocytes, PMNs, lymphocytes, eosinophils, and neutrophils), serum uric acid (sUA), ESR, and CRP were recorded.

### 2.3. Statistical Analysis

Database management and statistical analyses were performed in SPSS 18.0. Quantitative variables are presented as the means ± standard deviations (SDs), and categorical variables are indicated as percentages (%). Differences in continuous variables were compared with Student's *t*-test. Categorical variables were compared with the *χ*^2^ test. Receiver operating characteristic (ROC) curves were generated to evaluate the diagnostic value of the variables for gout patients and normal sUA gout patients compared with RA, axSpA, and OA patients. A *P* value < 0.05 was accepted as significant.

## 3. Results

### 3.1. Basic Characteristics of the Participants

The age of gout patients was older than that of axSpA patients and younger than those of RA and OA patients (*P* < 0.05). The synovial fluid WBC, PBMC, monocyte, PMN, and neutrophil counts in gout patients were higher than those in OA patients (*P* < 0.05). The synovial fluid PBMC and lymphocyte counts in gout patients were lower than those in RA and axSpA patients (*P* < 0.05). The sUA levels of gout patients were higher than those of RA, axSpA, and OA patients (*P* < 0.05). The ESR and CRP levels of gout, RA, and axSpA patients were higher than those of OA patients (*P* < 0.05) (Table 1).

### 3.2. ROC Curves Were Used to Evaluate the Diagnostic Value of Synovial Fluid Cell Counts for Gout Patients

**Figure 1:**
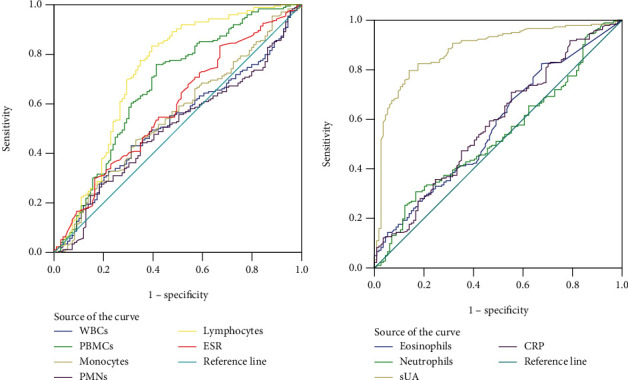
ROC curves were used to evaluate the diagnostic value of synovial fluid cell counts for gout patients.

ROC curves were used to evaluate the diagnostic value of synovial fluid WBCs, PBMCs, monocytes, PMNs, lymphocytes, eosinophils, and neutrophils for gout patients compared with RA, axSpA, and OA patients. The results showed that the AUC values of lymphocytes and sUA for gout patients were 0.728 (95% CI: 0.662–0.793) and 0.881 (95% CI: 0.840–0.922), respectively, which were higher than those of other variables. The optimal cutoff value of lymphocytes for gout patients was 1.362, with a Youden index of 0.439, a sensitivity of 83.3%, and a specificity of 60.6% (Table 2 and Figure 1).

### 3.3. ROC Curves Were Used to Evaluate the Diagnostic Value of Synovial Fluid Cell Counts for Normal sUA Gout Patients

**Figure 2:**
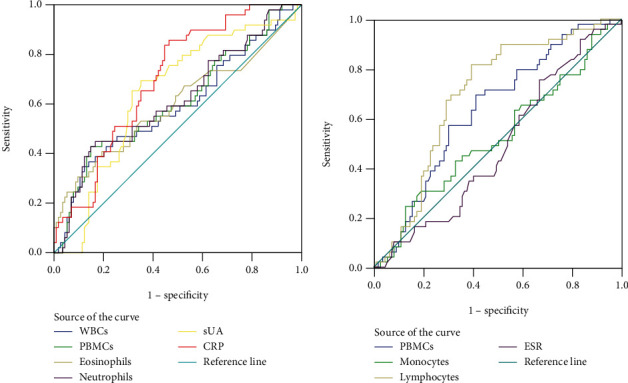
ROC curves were used to evaluate the diagnostic value of synovial fluid cell counts for normal sUA gout patients.

ROC curves were used to evaluate the diagnostic value of synovial fluid WBCs, PBMCs, monocytes, PMNs, lymphocytes, eosinophils, and neutrophils for normal sUA gout patients compared with RA, axSpA, and OA patients. The results showed that the AUC values of lymphocytes, sUA, and CRP for normal sUA gout were 0.694 (95% CI: 0.609–0.779), 0.643 (95% CI: 0.553–0.733), and 0.700 (95% CI: 0.619–0.781), respectively, which were higher than those of the other variables. The optimal cutoff value of lymphocytes for normal sUA gout patients was 1.362, with a Youden index of 0.422, a sensitivity of 81.6%, and a specificity of 60.6% (Table 3 and Figure 2).

## 4. Discussion

The mechanism involved in MSU crystal-induced inflammation of gout is complex, and few studies have been performed to describe the characteristics of synovial fluid cell counts in gout patients. In our study, we found that the synovial fluid cell counts of gout patients were different from those of RA, axSpA, and OA patients. Synovial fluid lymphocytes had a higher diagnostic value for gout than RA, axSpA, and OA.

MSU crystal identification of synovial fluid provides etiological proof of gout and is considered the gold standard for diagnosis. However, MSU crystals sometimes cannot be detected, especially in patients with a first episode of gout. Calculation of synovial fluid cell counts using a Sysmex XP-300 is an easy, inexpensive, and routine examination technique that provides information about the immune cells in synovial fluid, including WBCs, PBMCs, PMNs, monocytes, lymphocytes, eosinophils, and neutrophils [17]. It has been reported that the number of leukocytes and the percentage of PMNs in synovial fluid in the acute phase of gout patients were higher than those in the remission phase [5]. In our study, we found that the synovial fluid WBC, PBMC, monocyte, PMN, and neutrophil counts in gout patients were higher than those in OA patients (*P* < 0.05), while the synovial fluid PBMC and lymphocyte counts in gout patients were lower than those in RA and axSpA patients (*P* < 0.05). Vaidya et al. found that the synovial fluid total cells of gout patients were higher than those of OA and AS patients and lower than those of RA patients [18], similar to our studies.

To further explore the diagnostic value of synovial fluid cell counts for gout, ROC curves were generated and compared with RA, axSpA, and OA. The results indicated that the AUC values of lymphocytes and sUA for gout patients were 0.728 and 0.881, respectively, which were higher than those of other variables. The optimal cutoff value of lymphocytes for gout patients was 1.362, with a Youden index of 0.439, a sensitivity of 83.3%, and a specificity of 60.6%. That is, synovial fluid lymphocytes can distinguish gout from RA, axSpA, and OA. In addition, sUA can decrease and be normal during gout attacks, misleading unaware clinicians. Therefore, whether synovial fluid lymphocytes can distinguish normal sUA gout patients on attacks from RA, axSpA, and OA patients is worth further exploration. In our study, gout patients were divided into normal sUA gout patients and high sUA gout patients according to the sUA level during attack. The ROC curve results showed that the AUCs of lymphocytes, sUA, and CRP for normal sUA gout patients were 0.694, 0.643, and 0.700, respectively, which were higher than those of RA, axSpA, and OA patients. The optimal cutoff value of lymphocytes for normal sUA gout patients was 1.362, with a Youden index of 0.422, a sensitivity of 81.6%, and a specificity of 60.6%. This is the first study to find such a high diagnostic value biomarker for gout from synovial fluid.

Our experiments have some limitations. First, this was a single-center study with a small number of cases. Second, the relationship between blood cell counts and synovial fluid cell counts was not evaluated. Therefore, further studies are needed to explore the characterization of synovial fluid cell counts in gout patients.

In conclusion, the present study demonstrated that the synovial fluid cell counts of gout patients were different from those of RA, axSpA, and OA patients. Synovial fluid lymphocytes had a higher diagnostic value for gout than for RA, axSpA, and OA. Synovial fluid lymphocytes may be a reliable, cost-effective, and novel potential biomarker for gout.

## Figures and Tables

**Table 1:** Basic characteristics of the participants.

	Gout (*n* = 185)	RA (*n* = 64)	axSpA (*n* = 26)	OA (*n* = 24)	*P* value
Age (years)	48.58 ± 15.58	56.19 ± 12.39^∗^	32.96 ± 15.19^∗^^#^	69.63 ± 12.43^∗^^#&^	<0.001
Gender (male/female)	176/9	11/53	21/5	8/16	<0.001
WBCs (×10^9^/L)	18.58 ± 22.94	22.24 ± 20.87	15.52 ± 15.03	3.03 ± 5.59^∗^^#&^	0.002
PBMCs (×10^9^/L)	1.85 ± 1.99	3.68 ± 2.43^∗^	3.85 ± 3.34^∗^	0.74 ± 1.01^∗^^#&^	<0.001
Monocytes (×10^9^/L)	1.02 ± 1.59	1.24 ± 1.11	1.34 ± 1.52	0.29 ± 0.37^∗^^#&^	0.030
PMNs (×10^9^/L)	16.77 ± 21.51	18.57 ± 19.32	15.75 ± 24.17	2.30 ± 5.00^∗^^#&^	0.008
Lymphocytes (×10^9^/L)	0.80 ± 0.83	2.43 ± 1.76^∗^	2.50 ± 2.04^∗^	0.45 ± 0.80^#&^	<0.001
Eosinophils (×10^9^/L)	1.32 ± 3.75	0.56 ± 0.93	0.11 ± 0.17	0.49 ± 1.85	0.098
Neutrophils (×10^9^/L)	16.42 ± 21.16	18.82 ± 20.89	11.13 ± 14.23	2.23 ± 4.87^∗^^#^	0.003
sUA (*μ*M)	497.92 ± 132.24	299.31 ± 97.91^∗^	351.81 ± 118.93^∗^	333.38 ± 75.19^∗^	<0.001
ESR (mm/h)	61.02 ± 37.68	82.42 ± 32.87^∗^	68.12 ± 36.25	42.34 ± 35.91^∗^^#&^	<0.001
CRP (mg/L)	56.52 ± 45.64	44.01 ± 35.27^∗^	65.49 ± 39.85^#^	22.11 ± 40.65^∗^^#&^	<0.001

^∗^*P* < 0.05 vs. gout group, ^#^*P* < 0.05 vs. RA group, ^&^*P* < 0.05 vs. axSpA group.

**Table 2:** ROC curves were used to evaluate the diagnostic value of synovial fluid cell counts for gout patients.

	AUC (95% CI)	*P* value	Optimal cutoff value	Youden index	Sensitivity	Specificity
WBCs	0.523 (0.455-0.591)	0.510				
PBMCs	0.670 (0.603-0.738)	<0.001				
Monocytes	0.551 (0.482-0.620)	0.150				
PMNs	0.502 (0.434-0.571)	0.945				
Lymphocytes	0.728 (0.662-0.793)	<0.001	1.362	0.439	83.3%	60.6%
Eosinophils	0.570 (0.503-0.637)	0.043				
Neutrophils	0.535 (0.468-0.602)	0.314				
sUA	0.881 (0.840-0.922)	<0.001	399.5	0.656	79.7%	86.0%
CRP	0.581 (0.514-0.648)	0.019				
ESR	0.585 (0.516-0.653)	0.017				

**Table 3:** ROC curves were used to evaluate the diagnostic value of synovial fluid cell counts for normal sUA gout patients.

	AUC (95% CI)	*P* value	Optimal cutoff value	Youden index	Sensitivity	Specificity
WBCs	0.592 (0.492-0.692)	0.064				
PBMCs	0.632 (0.543-0.722)	0.008				
Monocytes	0.523 (0.423-0.622)	0.651				
PMNs	0.609 (0.511-0.706)	0.028				
Lymphocytes	0.694 (0.609-0.779)	<0.001	1.362	0.422	81.6%	60.6%
Eosinophils	0.596 (0.493-0.699)	0.052				
Neutrophils	0.617 (0.521-0.714)	0.018				
sUA	0.643 (0.553-0.733)	0.004	352.5	0.337	65.3%	68.4%
CRP	0.700 (0.619-0.781)	<0.001	36.45	0.392	85.7%	53.5%
ESR	0.516 (0.423-0.610)	0.744				

## Data Availability

The [data type] data used to support the findings of this study are available from the corresponding authors upon request.
